# Applying asymptotic methods to synthetic biology: Modelling the reaction kinetics of the mevalonate pathway

**DOI:** 10.1016/j.jtbi.2017.11.022

**Published:** 2018-02-14

**Authors:** Mohit P. Dalwadi, Marco Garavaglia, Joseph P. Webb, John R. King, Nigel P. Minton

**Affiliations:** a Synthetic Biology Research Centre, University of Nottingham, University Park, Nottingham NG7 2RD, UK; b Department of Molecular Biology and Biotechnology, University of Sheffield, Western Bank, Sheffield S10 2TN, UK; c School of Mathematical Sciences, University of Nottingham, University Park, Nottingham NG7 2RD, UK

**Keywords:** Asymptotic analysis, Metabolic pathways, Isoprenoid production

## Abstract

The mevalonate pathway is normally found in eukaryotes, and allows for the production of isoprenoids, a useful class of organic compounds. This pathway has been successfully introduced to *Escherichia coli*, enabling a biosynthetic production route for many isoprenoids. In this paper, we develop and solve a mathematical model for the concentration of metabolites in the mevalonate pathway over time, accounting for the loss of acetyl-CoA to other metabolic pathways. Additionally, we successfully test our theoretical predictions experimentally by introducing part of the pathway into *Cupriavidus necator*. In our model, we exploit the natural separation of time scales as well as of metabolite concentrations to make significant asymptotic progress in understanding the system. We confirm that our asymptotic results agree well with numerical simulations, the former enabling us to predict the most important reactions to increase isopentenyl diphosphate production whilst minimizing the levels of HMG-CoA, which inhibits cell growth. Thus, our mathematical model allows us to recommend the upregulation of certain combinations of enzymes to improve production through the mevalonate pathway.

## Highlights

• We investigate a kinetic model for the mevalonate pathway which includes inhibition effects and a sink of acetyl-CoA.
• Of the enzymes in the pathway, upregulating HMG-CoA reductase has the most significant positive effect on improving pathway efficiency.
• Upregulating pyruvate dehydrogenase complex and HMG-CoA synthase can also help, but only in conjunction with the upregulation of HMG-CoA reductase.
• We confirm our theoretical predictions by introducing the mevalonate pathway into Cupriavidus necator.

## 1 Introduction

Isoprenoids are a diverse class of naturally occurring organic chemicals found in all organisms. In plants, isoprenoids are the cause of many aromas and, in animals, isoprenoids form steroids and sterols. The wide range of isoprenoid products is one reason why the successful introduction of a viable isoprenoid pathway to *Escherichia coli* by Martin et al. (2003) (in this case to produce amorpha-4,11-diene, a pre-cursor to the antimalarial compound artemisinin) was a major breakthrough in synthetic biology. Since then, a significant amount of experimental work has been carried out to improve the yield from this pathway (see, for example, Anthony, Anthony, Nowroozi, Kwon, Newman, Keasling, 2009, Kizer, Pitera, Pfleger, Keasling, 2008, Newman, Marshall, Chang, Nowroozi, Paradise, Pitera, Newman, Keasling, 2006, Pitera, Paddon, Newman, Keasling, 2007, Tabata, Hashimoto, 2004).

There are two main pathways from pyruvate to isopentenyl diphosphate (IDP) and dimethylallyl diphosphate (DMADP), and these two products can react to make isoprenoid compounds. IDP and DMADP are essentially interchangeable due to the enzyme isopentenyl diphosphate isomerase that allows conversion between the two. The first pathway is known as the mevalonate pathway, and starts from acetyl coenzyme A (acetyl-CoA), mainly derived from pyruvate, which is converted to IDP via the key pathway intermediate mevalonate. The second pathway is known as the non-mevalonate or, alternatively, the 2-C-methyl-*D*-erythritol 4-phosphate/1-deoxy-*D*-xylulose 5-phosphate (MEP/DOXP) pathway, and also converts pyruvate to IDP. The mevalonate pathway was the first to be discovered, and occurs naturally in eukaryotes. The non-mevalonate pathway mainly occurs in bacteria (with some exceptions), and some plants.

**Fig. 1:**
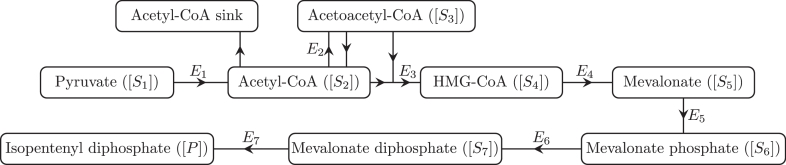
A schematic network diagram for the pathway we consider in this paper, where arrows denote the direction of the reactions. We only track the metabolites included in this Figure and, specifically, not any involved in the acetyl-CoA sink. Where we write *E_i_* (for i=1,…,7) next to a reaction arrow, this denotes a specific enzyme that controls the reaction. Hence, *E*_1_ corresponds to the pyruvate dehydrogenase complex (EC 1.2.4.1, EC 2.3.1.12, and EC 1.8.1.4), *E*_2_ corresponds to acetyl-CoA acetyltransferase (EC 2.3.1.9), *E*_3_ corresponds to HMG-CoA synthase (EC 2.3.3.10), *E*_4_ corresponds to HMG-CoA reductase (EC 1.1.1.34), *E*_5_ corresponds to mevalonate kinase (EC 2.7.1.36), *E*_6_ corresponds to phosphomevalonate kinase (EC 2.7.4.2), and *E*_7_ corresponds to mevalonate diphosphate decarboxylase (EC 4.1.1.33).

The reason for introducing the mevalonate pathway to *E. coli*, a bacterium that naturally expresses only the non-mevalonate pathway, is to bypass the natural negative feedback mechanisms in place that would ordinarily prevent the overproduction of isoprenoids. We are interested in mathematically modelling this mevalonate pathway, with the goal of understanding how to further modify the pathway by, for example, upregulating genes that control certain enzymes, in order to produce more IDP. The mevalonate pathway we will model comprises the following:

(1a) $$\begin{array}{cc} \text{Pyruvate} & \overset{k_{1}}{\to }\text{Acetyl-CoA}, \end{array}$$

(1b) $$\begin{array}{cc} \text{Acetyl-CoA} & \overset{k_{2}}{\underset{k_{-2}}{⇌}}\text{Acetoacetyl-CoA}, \end{array}$$

(1c) $$\begin{array}{cc} \text{Acetyl-CoA} & \overset{A}{\to }\phi , \end{array}$$

(1d) $$\begin{array}{cc} \text{Acetoacetyl-CoA}+\text{Acetyl-CoA} & \overset{k_{3}}{\to }\text{HMG-CoA}, \end{array}$$

(1e) $$\begin{array}{cc} \text{HMG-CoA} & \overset{k_{4}}{\to }\text{Mevalonate}, \end{array}$$

(1f) $$\begin{array}{cc} \text{Mevalonate} & \overset{k_{5}}{\to }\text{Mevalonate}\;\text{phosphate}, \end{array}$$

(1g) $$\begin{array}{cc} \text{Mevalonate}\;\text{phosphate} & \overset{k_{6}}{\to }\text{Mevalonate}\;\text{diphosphate}, \end{array}$$

(1h) $$\begin{array}{cc} \text{Mevalonate}\;\text{diphosphate} & \overset{k_{7}}{\to }\text{Isopentenyl}\;\text{diphosphate}, \end{array}$$

where (1c) represents the loss of acetyl-CoA to other metabolic pathways, such as the citric acid cycle or any pathways directly involved with fatty acid biosynthesis. Moreover, we will not keep track of any metabolites associated with this acetyl-CoA sink. We show a schematic of the pathway in Fig. 1.

In general, the aim of our model is to determine which reactions are the most important for IDP production without resorting to expensive and time-consuming experiments. We are also interested in determining which reactions have the most significant control over the levels of 3-hydroxy-3-methylglutaryl-coenzyme A (HMG-CoA), linked to the inhibition of cell growth due to its inhibition of fatty acid biosynthesis (Kizer et al., 2008). Specifically, we will be interested in maintaining low levels of HMG-CoA whilst increasing IDP production.

We make several key modelling assumptions to facilitate analysis of our system. Firstly, we assume that the formation rate of enzyme complex is much quicker than the rate of substrate consumption, and thus the reaction rates are governed by Michaelis–Menten-type laws, the specific form of which we obtain from the literature. We also consider a system that is well mixed and thus spatially independent. Additionally, we consider the case where pyruvate is instantaneously introduced to a system containing all of the relevant enzymes, but none of the intermediate metabolites, allowing for a cleaner mathematical analysis. We first investigate the case where pyruvate is continuously replenished and held at a constant concentration, then the case where pyruvate is never replenished. We show that the second case shares many similarities with the first until the pyruvate is depleted to a certain critical level, which we determine. Understanding these extreme cases allows us to determine the key reactions in this pathway, and to suggest targets for upregulation.

As is the case with many biological systems, there are many parameters in the system. Thus, a comprehensive understanding of the system using a purely experimental approach would be very time consuming. This reasoning also applies to investigating our mathematical model using a fully numerical approach, although the time taken to investigate the system would be shorter than the purely experimental approach. To get around this issue, we supplement and guide our numerical simulations by determining asymptotic approximations (see, for example, Hinch, 1991, Kevorkian, Cole, 2013, O’Malley, Jr., 2012) of the metabolite concentrations. This will enhance our physical insight into the underlying system and allow us to determine how the concentrations vary as functions of the experimental parameters. Moreover, this approach allows us to bypass the issue we have with the uncertainty in the parameters, as this method only requires an idea of the order of magnitude of each parameter.

Finally, to test our theoretical predictions, we introduce part of the mevalonate pathway (from acetyl-CoA to mevalonate) into *Cupriavidus necator* by transforming it with a plasmid harbouring the *mvaE* and *mvaS* genes from *Enterococcus faecalis* under the control of the P_BAD_ *L*-arabinose inducible promoter. The *mvaE* and *mvaS* genes code for the enzymes responsible for the conversion of acetyl-CoA to mevalonate (in the first half of the mevalonate pathway). We show that mevalonate can be produced by our bacterial chassis, and confirm that our experimental results are successfully predicted by our model.

The outline of this paper is as follows. We introduce a mathematical model to describe the nonlinear reaction kinetics in Section 2. We solve this system in Section 3, where we give both numerical and asymptotic solutions to describe the system behaviour. In that section, we consider the continuous replenishment of pyruvate case first, then we consider the no replenishment of pyruvate case. We discuss the experimental procedure and results in Section 4, and compare these results to our model predictions. We finish by discussing our results and comparing the two regimes in Section 5.

## 2 Model description

The dimensional system we consider is derived from Michaelis–Menten-type laws found in the literature, where each variable is defined in Table 1. The forms of each reaction rate are obtained from the corresponding reference in Table 2. The dimensional system is as follows

(2a) $$\begin{array}{cc} \frac{d[S_{1}]}{d\tau } & =-\frac{k_{1}E_{1}K_{1}^{i}\left[S_{1}\right]}{K_{1}^{i}\left(\left[S_{1}\right]+K_{1}^{M}\right)+\left[S_{1}\right]\left[S_{2}\right]}, \end{array}$$

(2b) $$\begin{array}{cc} \frac{d[S_{2}]}{d\tau } & =\frac{k_{1}E_{1}K_{1}^{i}\left[S_{1}\right]}{K_{1}^{i}\left(\left[S_{1}\right]+K_{1}^{M}\right)\;+\;\left[S_{1}\right]\left[S_{2}\right]}\;-\;\frac{k_{2}E_{2}\left[S_{2}\right]}{\left[S_{2}\right]+K_{2}^{M}}+\frac{k_{-2}E_{2}\left[S_{3}\right]}{\left[S_{3}\right]\;+\;K_{-2}^{M}}\;-\;A\left[S_{2}\right], \end{array}$$

(2c) $$\begin{array}{ccc} \frac{d[S_{3}]}{d\tau } & = & \frac{k_{2}E_{2}\left[S_{2}\right]}{\left[S_{2}\right]+K_{2}^{M}}-\frac{k_{-2}E_{2}\left[S_{3}\right]}{\left[S_{3}\right]+K_{-2}^{M}}\\ & & -\;\frac{k_{3}E_{3}K_{3}^{i}\left[S_{2}\right]\left[S_{3}\right]}{K_{3}^{i}\left[S_{2}\right]\left[S_{3}\right]+K_{3,a}^{M}\left[S_{3}\right]\left(\left[S_{3}\right]+K_{3}^{i}\right)+K_{3}^{i}K_{3,b}^{M}\left[S_{2}\right]}, \end{array}$$

(2d) $$\begin{array}{cc} \frac{d[S_{4}]}{d\tau } & =\frac{k_{3}E_{3}K_{3}^{i}\left[S_{2}\right]\left[S_{3}\right]}{K_{3}^{i}\left[S_{2}\right]\left[S_{3}\right]+K_{3,a}^{M}\left[S_{3}\right]\left(\left[S_{3}\right]+K_{3}^{i}\right)+K_{3}^{i}K_{3,b}^{M}\left[S_{2}\right]}-\frac{k_{4}E_{4}\left[S_{4}\right]}{\left[S_{4}\right]+K_{4}^{M}}, \end{array}$$

(2e) $$\begin{array}{cc} \frac{d[S_{5}]}{d\tau } & =\frac{k_{4}E_{4}\left[S_{4}\right]}{\left[S_{4}\right]+K_{4}^{M}}-\frac{k_{5}E_{5}\left[S_{5}\right]}{\left[S_{5}\right]+K_{5}^{M}}, \end{array}$$

(2f) $$\begin{array}{cc} \frac{d[S_{6}]}{d\tau } & =\frac{k_{5}E_{5}\left[S_{5}\right]}{\left[S_{5}\right]+K_{5}^{M}}-\frac{k_{6}E_{6}\left[S_{6}\right]}{\left[S_{6}\right]+K_{6}^{M}}, \end{array}$$

(2g) $$\begin{array}{cc} \frac{d[S_{7}]}{d\tau } & =\frac{k_{6}\left[S_{6}\right]}{\left[S_{6}\right]+K_{6}^{M}}-\frac{k_{7}E_{7}\left[S_{7}\right]}{\left[S_{7}\right]+K_{7}^{M}}, \end{array}$$

(2h) $$\begin{array}{cc} \frac{d[P]}{d\tau } & =\frac{k_{7}E_{7}\left[S_{7}\right]}{\left[S_{7}\right]+K_{7}^{M}}. \end{array}$$

**Table 1:** Dimensional and dimensionless variable definitions.

Original variable	Description	Nondimensionalisation
[*S*_1_]	Pyruvate	$\left[S_{1}\right]=S_{0}S_{1}$
[*S*_2_]	Acetyl CoA	$\left[S_{2}\right]=S_{0}S_{2}$
[*S*_3_]	Acetoacetyl-CoA	$\left[S_{3}\right]=S_{0}S_{3}$
[*S*_4_]	HMG-CoA	$\left[S_{4}\right]=S_{0}S_{4}$
[*S*_5_]	Mevalonate	$\left[S_{5}\right]=S_{0}S_{5}$
[*S*_6_]	Mevalonate phosphate	$\left[S_{6}\right]=S_{0}S_{6}$
[*S*_7_]	Mevalonate diphosphate	$\left[S_{7}\right]=S_{0}S_{7}$
[*P*]	Isopentenyl diphosphate	$\left[P\right]=S_{0}P$
*τ*	Time	$\tau =(S_{0}/k_{1}E_{1})t$

**Table 2:** Parameters. We use the value $S_{0}=1\;\mathrm{mM},$ and assume that $E_{i}=E_{j}$ for i,j=1,…,7. Different values of *E_j_* can be considered by varying the appropriate dimensionless parameter. We introduce the small dimensionless parameter ɛ=0.01, to formally account for the large difference in magnitude between parameters, and choose $\bar{A}=1$ in the simulations, as there is a distinguished asymptotic limit when $\bar{A}=O\left(1\right)$.

Dimensional	Organism	Range
$k_{1}=10\;s^{-1}$	*Saccharomyces cerevisiae* (Kresze, Ronft, 1981, Kresze, Ronft, 1981)	4 – $30\;s^{-1}$ (Kresze, Ronft, 1981, Kresze, Ronft, 1981, Snoep, De Mattos, Starrenburg, Hugenholtz, 1992)
$k_{2}=200\;s^{-1}$	*Enterococcus faecalis* (Hedl et al., 2002)	10 – $260\;s^{-1}$ (Hedl, Sutherlin, Wilding, Mazzulla, McDevitt, Lane, Burgner, Lehnbeuter, Stauffacher, Gwynn, Rodwell, 2002, Matsumoto, Tanaka, Watanabe, Motohashi, Ikeda, Tobitani, Yao, Tanaka, Taguchi, 2013, Okamura, Tomita, Sawa, Nishiyama, Kuzuyama, 2010)
$k_{-2}=3000\;s^{-1}$	*Enterococcus faecalis* (Hedl et al., 2002)	80 – $3600\;s^{-1}$ (Hedl, Sutherlin, Wilding, Mazzulla, McDevitt, Lane, Burgner, Lehnbeuter, Stauffacher, Gwynn, Rodwell, 2002, Reddick, Williams, 2008)
$k_{3}=6\;s^{-1}$	*Saccharomyces cerevisiae* (Middleton, 1972)	0.5 – $14\;s^{-1}$ (Middleton, 1972, Nagegowda, Mee-Len, et al., 2004, Sutherlin, Hedl, Sanchez-Neri, Burgner, Stauffacher, Rodwell, 2002)
$k_{4}=10\;s^{-1}$	*Enterococcus faecalis* (Hedl et al., 2002)	1 – $20\;s^{-1}$ (Ching, Davies, Hardie, 1996, Hedl, Sutherlin, Wilding, Mazzulla, McDevitt, Lane, Burgner, Lehnbeuter, Stauffacher, Gwynn, Rodwell, 2002, Ma, Garcia, Redding-Johanson, Friedland, Chan, Batth, Haliburton, Chivian, Keasling, Petzold, Lee, R, 2011)
$k_{5}=20\;s^{-1}$	*Methanosarcina mazei* (Primak et al., 2011)	4 – $40\;s^{-1}$ (Huang, Scott, Bennett, 1999, Primak, Du, Miller, Wells, Nielsen, Weyler, Beck, 2011)
$k_{6}=4\;s^{-1}$	*Saccharomyces cerevisiae* (Garcia and Keasling, 2014)	2 – $6\;s^{-1}$ (Garcia, Keasling, 2014, Voynova, Rios, Miziorko, 2004)
$k_{7}=1\;s^{-1}$	*Saccharomyces cerevisiae* (Krepkiy and Miziorko, 2004)	0.1 – $5.5\;s^{-1}$ (Krepkiy, Miziorko, 2004, Qiu, Gao, Guo, Qiao, Li, 2007)
$K_{1}^{M}=0.65\;\mathrm{mM}$	*Saccharomyces cerevisiae* (Kresze and Ronft, 1981a)	0.13 – 1 mM (Kresze, Ronft, 1981, Pronk, Yde Steensma, van Dijken, 1996, Snoep, De Mattos, Starrenburg, Hugenholtz, 1992)
$K_{1}^{i}=0.014\;\mathrm{mM}$	*Saccharomyces cerevisiae* (Kresze and Ronft, 1981a)	0.014 – 0.018 mM (Kresze, Ronft, 1981, Pronk, Yde Steensma, van Dijken, 1996)
$K_{2}^{M}=1\;\mathrm{mM}$	*Enterococcus faecalis* (Hedl et al., 2002)	0.06 – 1.2 mM (Hedl, Sutherlin, Wilding, Mazzulla, McDevitt, Lane, Burgner, Lehnbeuter, Stauffacher, Gwynn, Rodwell, 2002, Matsumoto, Tanaka, Watanabe, Motohashi, Ikeda, Tobitani, Yao, Tanaka, Taguchi, 2013, Okamura, Tomita, Sawa, Nishiyama, Kuzuyama, 2010)
$K_{-2}^{M}=0.01\;\mathrm{mM}$	*Enterococcus faecalis* (Hedl et al., 2002)	0.01 – 0.09 mM (Hedl, Sutherlin, Wilding, Mazzulla, McDevitt, Lane, Burgner, Lehnbeuter, Stauffacher, Gwynn, Rodwell, 2002, Reddick, Williams, 2008)
$K_{3,a}^{M}=0.015\;\mathrm{mM}$	*Saccharomyces cerevisiae* (Middleton, 1972)	0.01 – 0.04 mM (Middleton, 1972, Nagegowda, Mee-Len, et al., 2004, Sutherlin, Hedl, Sanchez-Neri, Burgner, Stauffacher, Rodwell, 2002)
$K_{3,b}^{M}=0.003\;\mathrm{mM}$	*Saccharomyces cerevisiae* (Middleton, 1972)	0.0001 – 0.01 mM (Middleton, 1972, Nagegowda, Mee-Len, et al., 2004)
$K_{3}^{i}=0.01\;\mathrm{mM}$	*Saccharomyces cerevisiae* (Middleton, 1972)	0.008 – 0.02 mM (Middleton, 1972, Nagegowda, Mee-Len, et al., 2004)
$K_{4}^{M}=0.02\;\mathrm{mM}$	*Enterococcus faecalis* (Hedl et al., 2002)	0.015 – 0.065 mM (Ching, Davies, Hardie, 1996, Hedl, Sutherlin, Wilding, Mazzulla, McDevitt, Lane, Burgner, Lehnbeuter, Stauffacher, Gwynn, Rodwell, 2002, Ma, Garcia, Redding-Johanson, Friedland, Chan, Batth, Haliburton, Chivian, Keasling, Petzold, Lee, R, 2011)
$K_{5}^{M}=0.1\;\mathrm{mM}$	*Methanosarcina mazei* (Primak et al., 2011)	0.06 – 0.24 mM (Huang, Scott, Bennett, 1999, Primak, Du, Miller, Wells, Nielsen, Weyler, Beck, 2011)
$K_{6}^{M}=0.9\;\mathrm{mM}$	*Saccharomyces cerevisiae* (Garcia and Keasling, 2014)	0.004 – 0.9 mM (Garcia, Keasling, 2014, Voynova, Rios, Miziorko, 2004)
$K_{7}^{M}=0.2\;\mathrm{mM}$	*Saccharomyces cerevisiae* (Krepkiy and Miziorko, 2004)	0.03 – 0.9 mM (Krepkiy, Miziorko, 2004, Qiu, Gao, Guo, Qiao, Li, 2007)
$A\;[s^{-1}]$		
Dimensionless parameters		
${\bar{k}}_{2}=ɛk_{2}E_{2}/k_{1}E_{1}=0.2$	${\bar{K}}_{1}^{M}=K_{1}^{M}/S_{0}=0.65$	${\bar{K}}_{4}^{M}=K_{4}^{M}/ɛS_{0}=2$
${\bar{k}}_{-2}=ɛk_{-2}E_{2}/k_{1}E_{1}=3$	${\bar{K}}_{1}^{i}=K_{1}^{i}/ɛS_{0}=1.4$	${\bar{K}}_{5}^{M}=K_{5}^{M}/S_{0}=0.1$
${\bar{k}}_{3}=k_{3}E_{3}/k_{1}E_{1}=0.6$	${\bar{K}}_{2}^{M}=K_{2}^{M}/S_{0}=1$	${\bar{K}}_{6}^{M}=K_{6}^{M}/S_{0}=0.9$
${\bar{k}}_{4}=k_{4}E_{4}/k_{1}E_{1}=1$	${\bar{K}}_{-2}^{M}=K_{-2}^{M}/ɛS_{0}=1$	${\bar{K}}_{7}^{M}=K_{7}^{M}/S_{0}=0.2$
${\bar{k}}_{5}=k_{5}E_{5}/k_{1}E_{1}=2$	${\bar{K}}_{3,a}^{M}=K_{3,a}^{M}/ɛS_{0}=1.5$	$\bar{A}=AS_{0}/k_{1}E_{1}=1$
${\bar{k}}_{6}=k_{6}E_{6}/k_{1}E_{1}=0.4$	${\bar{K}}_{3,b}^{M}=K_{3,b}^{M}/ɛS_{0}=0.3$	
${\bar{k}}_{7}=k_{7}E_{7}/k_{1}E_{1}=0.1$	${\bar{K}}_{3}^{i}=K_{3}^{i}/ɛS_{0}=1$	

Most of the terms in the governing equations (2) are standard Michaelis–Menten reaction velocities, for which we obtain the relevant kinetic parameters from the references indicated in Table 2. The two modified Michaelis–Menten terms we include are for reactions (1a) and (1d), which describe different types of inhibition. In Kresze and Ronft (1981a), it is shown that acetyl-CoA has an inhibitory effect on (1a) which is uncompetitive with pyruvate. Therefore, for this reaction velocity we use the standard form for uncompetitive inhibition (Yung-Chi and Prusoff, 1973). In Middleton (1972), it is shown that acetoacetyl-CoA has an inhibitory effect on (1d) which is competitive with acetyl-CoA, and we therefore take this reaction velocity to have the standard form for competitive inhibition, as stated in Middleton (1972) and Yung-Chi and Prusoff (1973). The parameter *A* represents the total loss of acetyl-CoA to other metabolic pathways, for example, the citric acid cycle or fatty acid biosynthesis, and we assume that this occurs with first-order kinetics. While this parameter is difficult to measure experimentally, we will bypass this issue by considering a distinguished limit in the dimensionless system when we perform an asymptotic analysis. The parameters *E_i_*, where i=1,…,7, denote the enzyme concentrations for the reactions they control.

We use initial conditions that correspond to the scenario where pyruvate is instantaneously introduced to a system containing all of the relevant enzymes, but none of the intermediate metabolites. That is, we use $\left[S_{1}\right]\left(0\right)=S_{0},$ $\left[S_{2}\right]\left(0\right)=\left[S_{3}\right]\left(0\right)=\left[S_{4}\right]\left(0\right)=\left[S_{5}\right]\left(0\right)=\left[S_{6}\right]\left(0\right)=\left[S_{7}\right]\left(0\right)=\left[P\right]\left(0\right)=0$. Here, *S*_0_ represents the initial or typical level of pyruvate present in the system. As any given metabolite may already be present in the real-world system, our approach to the initial conditions is a modelling choice. That is, we choose to reduce the number of uncertain parameters in the system in order to facilitate a more simplified analysis of the system.

To nondimensionalize the system variables, we scale each dimensional metabolite concentration with *S*_0_, the initial concentration of pyruvate. Additionally, we scale time with *S*_0_/(*k*_1_*E*_1_), the characteristic time of the first reaction, which occurs between pyruvate and acetyl-CoA. We summarise these scalings in Table 1. To form dimensionless parameters, we first note that estimates of the kinetic parameters can vary significantly in different environments (Table 2). Given the uncertainty in the parameters, we seek to understand how the system behaves for different values of these parameters; we seek asymptotic solutions in terms of the system parameters, allowing us to explicitly determine how a variation in parameter values affects the system. We can explore how the system behaves as these parameters vary within an order of magnitude by first scaling each rate constant with the rate constant of the first reaction, each Michaelis constant with the initial pyruvate concentration, and each enzyme concentration with concentration of the first enzyme. Then, we use the typical dimensional values in Table 2 to introduce an artificial small dimensionless parameter ɛ=0.01 into the system, and write each dimensionless parameter as *c*ϵ^*j*^, where *c* is an *O*(1) parameter (between 0.1 and 10), and *j* is an integer. The resultant dimensionless parameters in our system are given in Table 2, and this approach allows us to interrogate the system using an asymptotic analysis (see, for example, Hinch, 1991, Kevorkian, Cole, 2013, O’Malley, Jr., 2012). Although, as is always the case with an asymptotic analysis, there may theoretically be an issue in equating terms with the same powers of ϵ when extreme *O*(1) parameters are multiplied together, we will show that our asymptotic and numerical results show excellent agreement, and thus the approach is reliable for this system.

Therefore, we nondimensionalize using the dimensionless variables defined in Table 1 and, using the dimensionless kinetic parameters defined in Table 2, we obtain the dimensionless system

(3a) $$\begin{array}{cc} \frac{dS_{1}}{dt} & =-\frac{ɛ{\bar{K}}_{1}^{i}S_{1}}{ɛ{\bar{K}}_{1}^{i}\left(S_{1}+{\bar{K}}_{1}^{M}\right)+S_{1}S_{2}}, \end{array}$$

(3b) $$\begin{array}{cc} \frac{dS_{2}}{dt} & =\frac{ɛ{\bar{K}}_{1}^{i}S_{1}}{ɛ{\bar{K}}_{1}^{i}\left(S_{1}+{\bar{K}}_{1}^{M}\right)+S_{1}S_{2}}-\frac{{\bar{k}}_{2}S_{2}}{ɛ\left(S_{2}+{\bar{K}}_{2}^{M}\right)}+\frac{{\bar{k}}_{-2}S_{3}}{ɛ\left(S_{3}+ɛ{\bar{K}}_{-2}^{M}\right)}-\bar{A}S_{2}, \end{array}$$

(3c) $$\begin{array}{ccc} \frac{dS_{3}}{dt} & = & \frac{{\bar{k}}_{2}S_{2}}{ɛ\left(S_{2}+{\bar{K}}_{2}^{M}\right)}-\frac{{\bar{k}}_{-2}S_{3}}{ɛ\left(S_{3}+ɛ{\bar{K}}_{-2}^{M}\right)}\\ & & -\frac{{\bar{k}}_{3}{\bar{K}}_{3}^{i}S_{2}S_{3}}{{\bar{K}}_{3}^{i}S_{2}S_{3}+{\bar{K}}_{3,a}^{M}S_{3}\left(S_{3}+ɛ{\bar{K}}_{3}^{i}\right)+ɛ{\bar{K}}_{3}^{i}{\bar{K}}_{3,b}^{M}S_{2}}, \end{array}$$

(3d) $$\begin{array}{cc} \frac{dS_{4}}{dt} & =\frac{{\bar{k}}_{3}{\bar{K}}_{3}^{i}S_{2}S_{3}}{{\bar{K}}_{3}^{i}S_{2}S_{3}+{\bar{K}}_{3,a}^{M}S_{3}\left(S_{3}+ɛ{\bar{K}}_{3}^{i}\right)+ɛ{\bar{K}}_{3}^{i}{\bar{K}}_{3,b}^{M}S_{2}}-\frac{{\bar{k}}_{4}S_{4}}{S_{4}+ɛ{\bar{K}}_{4}^{M}}, \end{array}$$

(3e) $$\begin{array}{cc} \frac{dS_{5}}{dt} & =\frac{{\bar{k}}_{4}S_{4}}{S_{4}+ɛ{\bar{K}}_{4}^{M}}-\frac{{\bar{k}}_{5}S_{5}}{S_{5}+{\bar{K}}_{5}^{M}}, \end{array}$$

(3f) $$\begin{array}{cc} \frac{dS_{6}}{dt} & =\frac{{\bar{k}}_{5}S_{5}}{S_{5}+{\bar{K}}_{5}^{M}}-\frac{{\bar{k}}_{6}S_{6}}{S_{6}+{\bar{K}}_{6}^{M}}, \end{array}$$

(3g) $$\begin{array}{cc} \frac{dS_{7}}{dt} & =\frac{{\bar{k}}_{6}S_{6}}{S_{6}+{\bar{K}}_{6}^{M}}-\frac{{\bar{k}}_{7}S_{7}}{S_{7}+{\bar{K}}_{7}^{M}}, \end{array}$$

(3h) $$\begin{array}{cc} \frac{dP}{dt} & =\frac{{\bar{k}}_{7}S_{7}}{S_{7}+{\bar{K}}_{7}^{M}}. \end{array}$$

In forming the dimensionless variables, we have used the physically plausible value of $S_{0}=1\;\mathrm{mM}$. Finally, the initial conditions become $S_{1}\left(0\right)=1,$ $S_{2}\left(0\right)=S_{3}\left(0\right)=S_{4}\left(0\right)=S_{5}\left(0\right)=S_{6}\left(0\right)=S_{7}\left(0\right)=P\left(0\right)=0$.

We solve this system for two cases. The first case is where (3a) does not hold and we instead impose *S*_1_(*t*) ≡ 1. This corresponds to the scenario where pyruvate is continuously replenished and held at a constant value. The second case is where (3a) does hold, and we will show that the two systems are equivalent for t=O(1). In both cases, we are interested in determining how to maximise the production of IDP whilst minimizing the levels of HMG-CoA, linked to the inhibition of cell growth.

## 3 Solutions

### 3.1 Numerical results

**Fig. 2:**
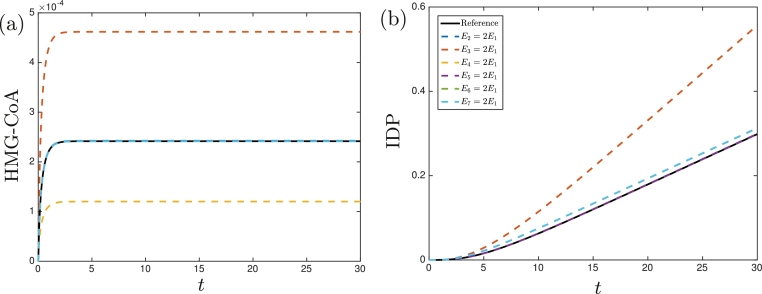
The numerically determined concentrations of (a) HMG-CoA and (b) IDP in the continuous replenishment of pyruvate case. The solid black lines denote the solutions using the reference parameter values given in Table 2 with $E_{i}=E_{j}$ for *i* ≠ *j*, and the dashed lines denote the solutions when a particular enzyme is over-expressed. The solutions when *E*_2_, *E*_5_, *E*_6_, or *E*_7_ are doubled are near identical to the reference concentration in (a). The solutions when *E*_2_, *E*_4_, or *E*_5_ are doubled are near identical to the reference concentration in (b), and the solutions when *E*_6_ or *E*_7_ are doubled are near identical to each other.

**Fig. 3:**
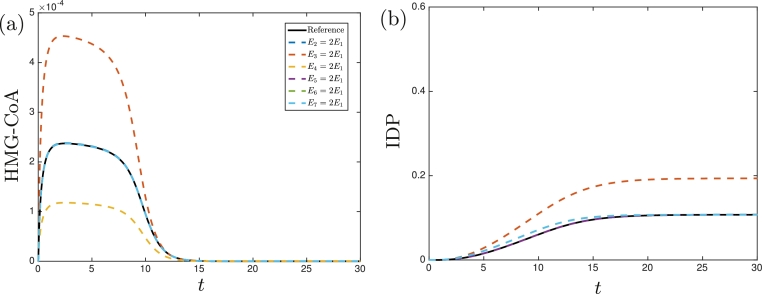
The numerically determined concentrations of (a) HMG-CoA and (b) IDP in the no replenishment of pyruvate case. The solid black lines denote the solutions using the parameter values given in Table 2 with $E_{i}=E_{j}$ for *i* ≠ *j*, and the dashed lines denote the solutions when a particular enzyme is over-expressed. The solutions when *E*_2_, *E*_5_, *E*_6_, or *E*_7_ are doubled are near identical to the reference concentration in (a). The solutions when *E*_2_, *E*_4_, or *E*_5_ are doubled are near identical to the reference concentration in (b), and the solutions when *E*_6_ or *E*_7_ are doubled are near identical to each other. We see that the solutions in this no replenishment of pyruvate case are very similar to the solutions in the continuous replenishment of pyruvate case until *t* ≈ 2 for HMG-CoA and until *t* ≈ 12 for IDP.

We solve the system presented in §2 numerically, using ode15s in MATLAB with a relative tolerance of $10^{-14}$. We use the parameter values given in Table 2 for the continuous replenishment of pyruvate (Fig. 2) and the no replenishment of pyruvate (Fig. 3) cases. In each case, we are also able to model the over-expression of an enzyme by increasing the dimensionless turnover numbers (the parameters denoted by a lower-case *k* with a subscript) given in Table 2.

Although the dynamics for each case are different, we can observe general trends. Most notably, we see that increasing *E*_3_ increases both the maximum levels of HMG-CoA and of IDP production, whereas increasing *E*_4_ decreases the maximum levels of HMG-CoA but has a negligible affect on IDP production. Comparing the continuously and never replenished pyruvate cases, we see that the initial dynamics appear to be similar between cases for the same parameter values (until *t* ≈ 2 for HMG-CoA and *t* ≈ 12 for IDP), but the dynamics diverge after a longer time. In the continuous replenishment case, the concentration of HMG-CoA and the production rate of IDP tends to a constant value whereas, in the no replenishment case, the concentration of HMG-CoA increases to a maximum level before decreasing, and the concentration of IDP tends to a constant value. To understand these phenomena in more detail, and to determine how we can increase IDP production whilst minimizing the maximum levels of (cell growth inhibiting) HMG-CoA in terms of the parameter values, we now perform an asymptotic analysis.

### 3.2 Asymptotic results

#### 3.2.1 Continuous replenishment of pyruvate

Here, we consider the system (3b–h), and impose ${\bar{S}}_{1}\left(t\right)\equiv 1$. As the sink reaction (1c) is an amalgamation of all sinks of acetyl-CoA (*S*_2_), it is difficult to obtain accurate estimates of $\bar{A}$. We proceed by assuming the distinguished limit $\bar{A}=O\left(1\right),$ and we discuss the further limit $\bar{A}=O\left(1/ɛ\right)$ in Appendix A. In pursuing an asymptotic analysis (see, for example, Hinch, 1991, Kevorkian, Cole, 2013, O’Malley, Jr., 2012) for small ϵ, we make the following scalings: $\left(S_{2},S_{5},S_{6},S_{7},P\right)={ɛ}^{1/2}\left({\bar{S}}_{2},{\bar{S}}_{5},{\bar{S}}_{6},{\bar{S}}_{7},\bar{P}\right),$ $\left(S_{3},S_{4}\right)={ɛ}^{3/2}\left({\bar{S}}_{3},{\bar{S}}_{4}\right)$ and we obtain the t=O(1) governing equations

(4a) $$\begin{array}{ccc} ɛ\frac{d{\bar{S}}_{2}}{dt} & = & \frac{ɛ{\bar{K}}_{1}^{i}{\bar{S}}_{1}}{{ɛ}^{1/2}{\bar{K}}_{1}^{i}\left({\bar{S}}_{1}+{\bar{K}}_{1}^{M}\right)+{\bar{S}}_{1}{\bar{S}}_{2}}-\frac{{\bar{k}}_{2}{\bar{S}}_{2}}{{ɛ}^{1/2}{\bar{S}}_{2}+{\bar{K}}_{2}^{M}}\\ & & +\frac{{\bar{k}}_{-2}{\bar{S}}_{3}}{{ɛ}^{1/2}{\bar{S}}_{3}+{\bar{K}}_{-2}^{M}}-ɛ\bar{A}{\bar{S}}_{2}, \end{array}$$

(4b) $$\begin{array}{cc} {ɛ}^{2}\frac{d{\bar{S}}_{3}}{dt} & =\frac{{\bar{k}}_{2}{\bar{S}}_{2}}{{ɛ}^{1/2}{\bar{S}}_{2}+{\bar{K}}_{2}^{M}}-\frac{{\bar{k}}_{-2}{\bar{S}}_{3}}{{ɛ}^{1/2}{\bar{S}}_{3}+{\bar{K}}_{-2}^{M}}\\ & \;-\frac{ɛ{\bar{k}}_{3}{\bar{K}}_{3}^{i}{\bar{S}}_{2}{\bar{S}}_{3}}{{ɛ}^{1/2}{\bar{K}}_{3}^{i}{\bar{S}}_{2}{\bar{S}}_{3}+ɛ{\bar{K}}_{3,a}^{M}{\bar{S}}_{3}\left({ɛ}^{1/2}{\bar{S}}_{3}+{\bar{K}}_{3}^{i}\right)+{\bar{K}}_{3}^{i}{\bar{K}}_{3,b}^{M}{\bar{S}}_{2}}, \end{array}$$

(4c) $$\begin{array}{cc} ɛ\frac{d{\bar{S}}_{4}}{dt} & =\frac{{\bar{k}}_{3}{\bar{K}}_{3}^{i}{\bar{S}}_{2}{\bar{S}}_{3}}{{ɛ}^{1/2}{\bar{K}}_{3}^{i}{\bar{S}}_{2}{\bar{S}}_{3}\;+\;ɛ{\bar{K}}_{3,a}^{M}{\bar{S}}_{3}\left({ɛ}^{1/2}{\bar{S}}_{3}\;+\;{\bar{K}}_{3}^{i}\right)\;+\;{\bar{K}}_{3}^{i}{\bar{K}}_{3,b}^{M}{\bar{S}}_{2}}\;-\;\frac{{\bar{k}}_{4}{\bar{S}}_{4}}{{ɛ}^{1/2}{\bar{S}}_{4}\;+\;{\bar{K}}_{4}^{M}}, \end{array}$$

(4d) $$\begin{array}{cc} \frac{d{\bar{S}}_{5}}{dt} & =\frac{{\bar{k}}_{4}{\bar{S}}_{4}}{{ɛ}^{1/2}{\bar{S}}_{4}+{\bar{K}}_{4}^{M}}-\frac{{\bar{k}}_{5}{\bar{S}}_{5}}{{ɛ}^{1/2}{\bar{S}}_{5}+{\bar{K}}_{5}^{M}}, \end{array}$$

(4e) $$\begin{array}{cc} \frac{d{\bar{S}}_{6}}{dt} & =\frac{{\bar{k}}_{5}{\bar{S}}_{5}}{{ɛ}^{1/2}{\bar{S}}_{5}+{\bar{K}}_{5}^{M}}-\frac{{\bar{k}}_{6}{\bar{S}}_{6}}{{ɛ}^{1/2}{\bar{S}}_{6}+{\bar{K}}_{6}^{M}}, \end{array}$$

(4f) $$\begin{array}{cc} \frac{d{\bar{S}}_{7}}{dt} & =\frac{{\bar{k}}_{6}{\bar{S}}_{6}}{{ɛ}^{1/2}{\bar{S}}_{6}+{\bar{K}}_{6}^{M}}-\frac{{\bar{k}}_{7}{\bar{S}}_{7}}{{ɛ}^{1/2}{\bar{S}}_{7}+{\bar{K}}_{7}^{M}}, \end{array}$$

(4g) $$\begin{array}{cc} \frac{d\bar{P}}{dt} & =\frac{{\bar{k}}_{7}{\bar{S}}_{7}}{{ɛ}^{1/2}{\bar{S}}_{7}+{\bar{K}}_{7}^{M}}. \end{array}$$

**Fig. 4:**
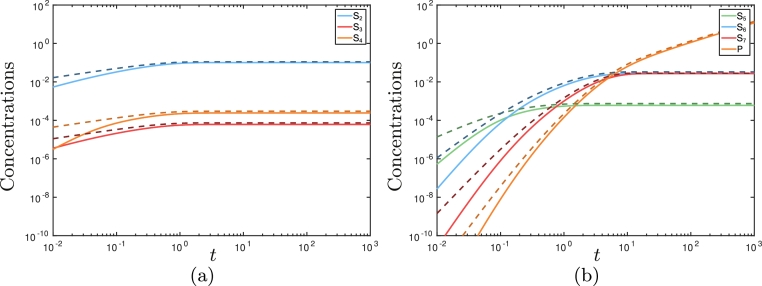
The numerical and asymptotic solutions for the metabolite concentrations in the continuous replenishment of pyruvate case. The solid light lines denote the numerical solutions, and the dashed darker lines denote the asymptotic solutions given in (6). We see good agreement between the numerical and asymptotic solutions for t=O(1), and the system attains its steady state solution in this region.

The leading-order version of (4) is given by

(5) $$\begin{array}{ccc} \frac{d{\bar{S}}_{2}}{dt} & = & \frac{{\bar{K}}_{1}^{i}}{{\bar{S}}_{2}}-v_{3}{\bar{S}}_{3}-\bar{A}{\bar{S}}_{2},\;0=v_{2}{\bar{S}}_{2}-v_{-2}{\bar{S}}_{3},\;0=v_{3}{\bar{S}}_{3}-v_{4}{\bar{S}}_{4},\\ \frac{d{\bar{S}}_{5}}{dt} & = & v_{4}{\bar{S}}_{4}-v_{5}{\bar{S}}_{5},\;\frac{d{\bar{S}}_{6}}{dt}=v_{5}{\bar{S}}_{5}-v_{6}{\bar{S}}_{6},\;\frac{d{\bar{S}}_{7}}{dt}=v_{6}{\bar{S}}_{6}-v_{7}{\bar{S}}_{7},\\ \frac{d\bar{P}}{dt} & = & v_{7}{\bar{S}}_{7}, \end{array}$$

where $v_{j}=k_{j}/K_{j}^{M}$ for j∈{2,−2,4,5,6,7}, and $v_{3}={\bar{k}}_{3}/{\bar{K}}_{3,b}^{M},$ and (5) is solved by

(6) $$\begin{array}{ccc} {\bar{S}}_{2} & = & {\left(\frac{{\bar{K}}_{1}^{i}\left(1-e^{-2\alpha t}\right)}{\alpha }\right)}^{1/2},\;{\bar{S}}_{3}=\frac{v_{2}}{v_{-2}}{\bar{S}}_{2},\;{\bar{S}}_{4}=\frac{v_{2}v_{3}}{v_{-2}v_{4}}{\bar{S}}_{2},\\ {\bar{S}}_{5} & = & \frac{v_{2}v_{3}}{v_{-2}}\int_{0}^{t}\;e^{v_{5}\left(s-t\right)}{\bar{S}}_{2}\left(s\right)\;ds,\\ {\bar{S}}_{6} & = & \frac{v_{2}v_{3}v_{5}}{v_{-2}}\int_{0}^{t}\;\frac{e^{v_{5}\left(s-t\right)}-e^{v_{6}\left(s-t\right)}}{v_{6}-v_{5}}{\bar{S}}_{2}\left(s\right)\;ds,\\ {\bar{S}}_{7} & = & \frac{v_{2}v_{3}v_{5}v_{6}}{v_{-2}}\int_{0}^{t}\;\frac{\left(v_{7}-v_{6}\right)e^{v_{5}\left(s-t\right)}-\left(v_{7}-v_{5}\right)e^{v_{6}\left(s-t\right)}+\left(v_{6}-v_{5}\right)e^{v_{7}\left(s-t\right)}}{\left(v_{7}-v_{6}\right)\left(v_{7}-v_{5}\right)\left(v_{6}-v_{5}\right)}{\bar{S}}_{2}\left(s\right)\;ds,\\ \bar{P} & = & \frac{v_{2}v_{3}v_{5}v_{6}v_{7}}{v_{-2}}\\ & & \times \int_{0}^{t}\;\frac{\frac{v_{7}-v_{6}}{v_{5}}\left(1-e^{v_{5}\left(s-t\right)}\right)-\frac{v_{7}-v_{5}}{v_{6}}\left(1-e^{v_{6}\left(s-t\right)}\right)+\frac{v_{6}-v_{5}}{v_{7}}\left(1-e^{v_{7}\left(s-t\right)}\right)}{\left(v_{7}-v_{6}\right)\left(v_{7}-v_{5}\right)\left(v_{6}-v_{5}\right)}{\bar{S}}_{2}\left(s\right)\;ds. \end{array}$$

where $\alpha =\bar{A}+v_{2}v_{3}/v_{-2}$. We see that these asymptotic solutions agree well with the numerical ones, except at early time where there is an additional asymptotic region that we do not consider (Fig. 4).

Although the leading-order solution for $\bar{P},$ given in (6), depends on parameters from almost all of the reactions, the dependence on *v*_5_, *v*_6_, and *v*_7_ is significantly reduced as *t* → ∞, and we may determine that the leading-order long-time behaviour in this regime is given by

(7) $$\begin{array}{c} \bar{P}∼\frac{v_{2}v_{3}}{v_{-2}}{\left(\frac{{\bar{K}}_{1}^{i}}{\alpha }\right)}^{1/2}t\;\text{as}\;t\to \infty . \end{array}$$

Hence, the reactions involving acetyl-CoA are the most important for the production of IDP. We can see that the numerical results shown in Fig. 4b agree with the analytic result that $\bar{P}$ tends to a linear function of time. We may also deduce the leading-order long-time behaviour of HMG-CoA in the form

(8) $$\begin{array}{c} {\bar{S}}_{4}\to \frac{v_{2}v_{3}}{v_{-2}v_{4}}{\left(\frac{{\bar{K}}_{1}^{i}}{\alpha }\right)}^{1/2}\;\text{as}\;t\to \infty . \end{array}$$

In dimensional terms, the long-time behaviour of the isopentenyl diphosphate (IDP) concentration is

(9a) $$\begin{array}{c} \left[P\right]∼\omega E_{3}{\left(\frac{E_{1}k_{1}K_{1}^{i}}{A+\omega E_{3}}\right)}^{1/2}\tau \;\text{as}\;\tau \to \infty , \end{array}$$

and the maximum level of HMG-CoA present in the system is

(9b) $$\begin{array}{c} \left[S_{4}\right]∼\frac{\omega E_{3}K_{4}^{M}}{k_{4}E_{4}}{\left(\frac{E_{1}k_{1}K_{1}^{i}}{A+\omega E_{3}}\right)}^{1/2}, \end{array}$$

where

(9c) $$\begin{array}{c} \omega =\frac{k_{2}k_{3}K_{-2}^{M}}{k_{-2}K_{2}^{M}K_{3,b}^{M}}. \end{array}$$

From the explicit results (9), we see that the long-time production of IDP depends on the enzyme concentrations *E*_1_ and *E*_3_, whereas the maximum concentration of HMG-CoA depends on the enzyme concentrations *E*_1_, *E*_3_, and *E*_4_. Recalling that our goal is to maximize IDP whilst minimizing HMG-CoA, and noting that the dependence on *E*_1_ and *E*_3_ is the same for both metabolites of interest, the only way our goal can be achieved by varying enzyme concentration is to significantly increase *E*_4_, so that it compensates for any increase in *E*_1_ or *E*_3_. That is, our model suggests that we should overexpress HMG-CoA reductase, the enzyme that catalyses the reaction from HMG-CoA to mevalonate. Importantly, we are able to deduce that this is the most significant leading-order effect, and this explicit result is possible due to our asymptotic analysis. The significance of this reaction is in agreement with Pitera et al. (2007), where it was shown that over-expression of HMG-CoA reductase alleviated the inhibition of cell growth, benefiting IDP production. Increasing *E*_3_, the enzyme that catalyses the reaction from acetoacetyl-CoA and acetyl-CoA to HMG-CoA would increase the levels of IDP produced by a single cell, but would also produce more HMG-CoA, which would reduce the number of cells in the system (though this is not formally taken into account by our model). The same is true of increasing *E*_1_, the enzyme that catalyses the reaction from pyruvate to acetyl-CoA, and decreasing *A*, any enzyme that catalyses reactions from acetyl-CoA to sinks of acetyl-CoA. Finally, we note that these results all agree with the numerical results in Fig. 2.

#### 3.2.2 No replenishment of pyruvate

We now consider the case where pyruvate can be depleted. Note that this case, unlike the previous, is singularly perturbed on the long timescale. From (3a), we must also consider the governing equation

(10) $$\begin{array}{c} \frac{d{\bar{S}}_{1}}{dt}=-\frac{{ɛ}^{1/2}{\bar{K}}_{1}^{i}{\bar{S}}_{1}}{{ɛ}^{1/2}{\bar{K}}_{1}^{i}\left({\bar{S}}_{1}+{\bar{K}}_{1}^{M}\right)+{\bar{S}}_{1}{\bar{S}}_{2}}, \end{array}$$

instead of imposing ${\bar{S}}_{1}\left(t\right)\equiv 1$. By seeking a power series representation for ${\bar{S}}_{1}$ in terms of the small parameter ϵ^1/2^, and using the solution (6 a) for ${\bar{S}}_{2},$ we obtain the following asymptotic solution to (10) for t=O(1):

(11) $$\begin{array}{c} {\bar{S}}_{1}\left(t\right)∼1-{\left(\frac{ɛ{\bar{K}}_{1}^{i}}{\alpha }\right)}^{1/2}\left(\alpha t+\log\left\{1+{\left[1-e^{-2\alpha t}\right]}^{1/2}\right\}\right)+O\left(ɛ\right). \end{array}$$

Importantly, we find that ${\bar{S}}_{1}\left(t\right)∼1+O\left({ɛ}^{1/2}\right)$ for t=O(1), and thus the no replenishment case is equivalent to the continuous replenishment case at leading order for t=O(1).

As suggested by (11), the *O*(1) decay of pyruvate occurs over the timescale $t=O({ɛ}^{-1/2})$. Further, as the dominant balances over this timescale do not change, the solution (11) allows us to deduce that the depletion of pyruvate occurs when

(12) $$\begin{array}{c} t^{*}=1/{\left(ɛ\alpha {\bar{K}}_{1}^{i}\right)}^{1/2}+O\left(1\right). \end{array}$$

For completion, we show the dynamics of this depletion, where the levels of pyruvate become exponentially small, in Appendix B. Using the depletion time (12) in the long-time IDP solution (7) and reverting back to the original unscaled version of IDP, we determine that the total amount of IDP produced in the no replenishment of pyruvate case is

(13) $$\begin{array}{c} \lim_{t\to \infty }P=\frac{v_{2}v_{3}}{\alpha v_{-2}}+O\left({ɛ}^{1/2}\right). \end{array}$$

Further, we may deduce that the maximum level of HMG-CoA in the system is again given by (8). In dimensional terms, the long-time behaviour of the isopentenyl diphosphate (IDP) concentration is

(14) $$\begin{array}{c} \left[P\right]∼\frac{\omega S_{0}E_{3}}{A+\omega E_{3}}\;\text{as}\;\tau \to \infty , \end{array}$$

and the maximum level of HMG-CoA present in the system is

(15) $$\begin{array}{c} \left[S_{4}\right]∼\frac{\omega E_{3}K_{4}^{M}}{k_{4}E_{4}}{\left(\frac{E_{1}k_{1}K_{1}^{i}}{A+\omega E_{3}}\right)}^{1/2}, \end{array}$$

where *ω* is defined in (9*c*).

Hence, our main conclusions from the continuous replenishment case are still valid for this case. For this no-replenishment-of-pyruvate case, we are also able to further determine that a lower value of $K_{1}^{i},$ perhaps achievable by introducing heterologous enzymes for the reaction (1a), would decrease the maximum amount of HMG-CoA present whilst having no significant effect on the total IDP produced.

## 4 Experimental validation

To validate the predictions made in this model, we carried out *in vivo* experiments in *Cupriavidus necator* H16, a gram-negative bacterium previously known as *Ralstonia eutropha. C. necator* is a facultative chemolithoautotrophic microorganism of relevant biotechnological interest since it can be exploited as a bacterial chassis for the production of chemicals. As proof of concept, we sought to introduce the upper part of the mevalonate pathway, leading to the production of isoprenoid precursors, into *C. necator*. To this end, *C. necator* H16 was transformed with a plasmid (pBBR1JW3) carrying the *mvaE* and *mvaS* genes from *E. faecalis* of the *L*-arabinose inducible promoter P_BAD_. These two genes code for the enzymes Acetyl-CoA acetyltransferase/HMG-CoA reductase (MvaE) and Hydroxymethylglutaryl-CoA synthase (MvaS), respectively. MvaE is a bifunctional enzyme that catalyses two reactions, from acetyl-CoA to acetoacetyl-CoA and from HMG-CoA to mevalonate, while MvaS converts acetoacetyl-CoA and acetyl-CoA to HMG-CoA. In our model, increasing the expression of MvaE corresponds to increasing *E*_2_ and *E*_4_, whereas increasing the expression of MvaS corresponds to increasing *E*_3_ (a schematic of the pathway is shown in Fig. 1). This plasmid provides a path from pyruvate to mevalonate (*S*_1_ to *S*_5_) within the modified *C. necator*.

### 4.1 Setting up of bacterial cultures for mevalonate production

Single colonies of *C. necator* H16/pBBR1-USERcassette1 and *C. necator* H16/pBBR1JW3 (pBBR1::*araC*/P_BAD_-*mvaES*) were used to inoculate 5ml of LB solution with 300µg/ml kanamycin (in 50ml tubes) and grown overnight at 30 °C with shaking (200 rpm). The following morning, the optical density (OD_600_) of the cultures was measured and normalised to OD${}_{600}=0.2$ in 100ml of LB solution with 300µg/ml kanamycin (in 500ml flasks). The bacterial cultures were then incubated at 30 °C with shaking (200 rpm) until they reached an OD_600_ of around 0.6 - 0.7. At this point, 1ml samples were collected from each culture, centrifuged at 14000 rpm for one minute and the cell pellets were stored at -20 °C to be used as pre-induction protein samples for SDS-PAGE. In addition, 1ml of 1% (w/v); 2% (w/v); or 20% (w/v) *L*-arabinose solutions were added to the corresponding cultures, thus obtaining *L*-arabinose final concentrations of 0.01%; 0.02%; and 0.2%, respectively. Following 18 hours of incubation at 30 °C with shaking (200 rpm), the OD_600_ of the cultures was measured. The bacterial cultures were then centrifuged (8000 rpm for five minutes), concentrated to OD${}_{600}=15$ in 1% fructose minimal medium (FMM) –NH_4_Cl with 300µg/ml kanamycin and transferred to 250ml flasks. After 24 hours of incubation at 30 °C with shaking (200 rpm), 500µl samples were taken from the cultures and centrifuged (14000 rpm for one minute). Supernatants were then analysed by HPLC to quantify the mevalonate titres produced by *C. necator* H16/pBBR1JW3 in response to the different *L*-arabinose concentrations taken into account.

### 4.2 Model comparison

We can use our model to predict the behaviour of the shorter pathway from pyruvate to mevalonate, by taking the limit ${\bar{k}}_{5},{\bar{k}}_{6},{\bar{k}}_{7}\to 0$. In this case, the results for [*S*_4_] remain the same and, in dimensional terms, the long-time results for [*S*_5_] are

(16) $$\begin{array}{c} \left[S_{5}\right]∼\omega E_{3}{\left(\frac{E_{1}k_{1}K_{1}^{i}}{A+\omega E_{3}}\right)}^{1/2}\tau \;\text{as}\;\tau \to \infty , \end{array}$$

for continuous replenishment of pyruvate and

(17) $$\begin{array}{c} \left[S_{5}\right]\to \frac{\omega S_{0}E_{3}}{A+\omega E_{3}}\;\text{as}\;\tau \to \infty , \end{array}$$

for no replenishment of pyruvate, where $\omega =k_{2}k_{3}K_{-2}^{M}/\left(k_{-2}K_{2}^{M}K_{3,b}^{M}\right),$ as previously defined in (9c). All variables are defined in §2. These are the same results our model predicts for [*P*], so our previous predictions for maximizing IDP while minimizing HMG-CoA will also be our current predictions for maximizing mevalonate while minimizing HMG-CoA in this experiment.

**Fig. 5:**
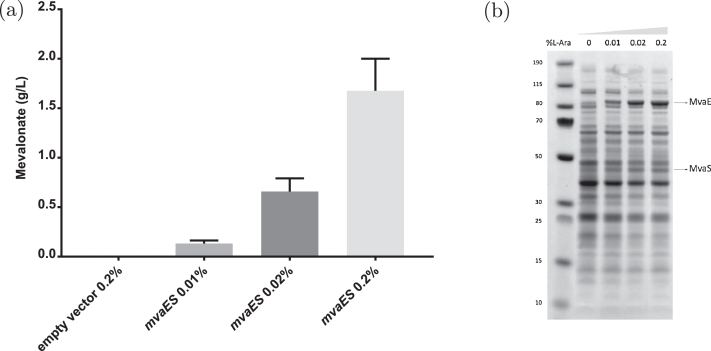
Experimental results for (a) mevalonate production by *C. necator* H16/pBBR1JW3 (pBBR1::*araC*/P_BAD_-*mvaES*) after 24 hours of growth in 1% (w/v) fructose minimal medium (FMM) in response to the following *L*-arabinose concentrations: 0.01% (w/v - grey bar); 0.02% (w/v - dark grey bar); and 0.2% (w/v - light grey bar). As a negative control, mevalonate production was assessed also with the *C. necator* H16/pBBR1-USERcassette1 strain (empty vector), that does not harbour the *mvaE* and *mvaS* genes. (b) SDS-PAGE of pre- (lane 1) and post-induction protein extracts of *C. necator* H16/pBBR1JW3. Expression of MvaE (86 KDa) and MvaS (42 KDa) was assessed following induction with 0.01% (lane 2); 0.02% (lane 3); and 0.2% (lane 4) *L*-arabinose. While MvaE expression levels increased with increasing *L*-arabinose concentrations, production of MvaS appeared to remain constant.

HPLC data show that increasing the concentration of the inducer *L*-arabinose leads to significant increments in the amount of mevalonate produced after 24 hours of growth in the presence of fructose as a carbon source (Fig. 5a). From the three levels of *L*-arabinose we considered, there appear to be diminishing returns on the effectiveness of increasing *L*-arabinose. From an SDS-PAGE, we see an increase in the expression of MvaE as the levels of *L*-arabinose are increased (Fig. 5b). However, production of MvaS appears to be constant as the levels of *L*-arabinose are increased. In our model, this corresponds to increasing *E*_2_ and *E*_4_ while keeping *E*_3_ (and all other enzyme levels) constant. As discussed above, our model predicts that this will decrease the maximum levels of HMG-CoA in the system, and thus produce more IDP. Hence, our model is verified by these experimental results.

## 5 Conclusions

We developed and solved a mathematical model for the kinetics of the mevalonate pathway, derived using generalised Michaelis–Menten kinetics and including the effect of a sink from acetyl-CoA into other metabolic pathways. We considered two extreme cases, namely where the pyruvate was continuously and never replenished. We used asymptotic analysis to gain physical insight into the system behaviour, allowing us to evaluate the effect of upregulating different reactions without resorting to an expensive parameter sweep. The system we considered here has eight dependent variables with 20 kinetic parameters. Our asymptotic analysis enabled us to give analytic expressions for each dependent variable in the continuous replenishment case, and to reduce the entire system to numerically solving a coupled nonlinear system of two dependent variables with one parameter in the never replenished case. We then validated our model by performing experiments that agreed with our predictions.

**Fig. 6:**
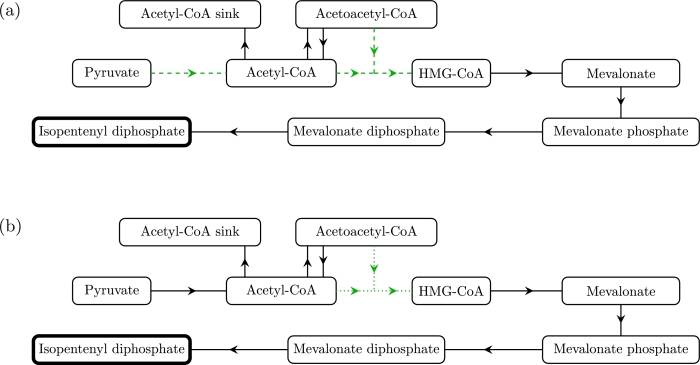
Schematic showing how overexpression of enzymes can affect IDP production in the cases of pyruvate being (a) continuously replenished and (b) never replenished. The green arrows denote that overexpressing the enzyme corresponding to that reaction results in greater production of IDP with diminishing returns. A dashed or dotted arrow denotes whether these diminishing returns are unbounded or bounded, respectively. (For interpretation of the references to colour in this figure legend, the reader is referred to the web version of this article.)

**Fig. 7:**
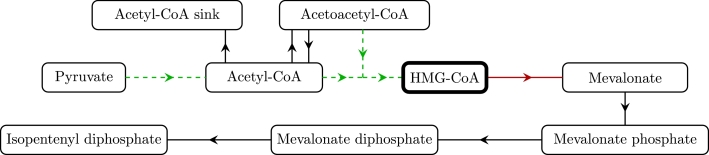
Schematic showing how overexpression of enzymes can affect levels of HMG-CoA. A green/red arrow denotes that overexpressing the enzyme corresponding to that reaction results in greater/lesser amounts of HMG-CoA, and a dashed arrow denotes that over-expression results in unbounded but diminishing returns. The results are the same for the cases of pyruvate being continuously and never replenished. (For interpretation of the references to colour in this figure legend, the reader is referred to the web version of this article.)

The main experimental goal is to maximise IDP production, whilst minimizing the maximum levels of HMG-CoA, a metabolite that is linked to the inhibition of cell growth due to its inhibition of fatty acid biosynthesis (Kizer et al., 2008). In terms of over-expressing enzymes, we see that over-expressing *E*_3_, the enzyme that catalyses the reaction from acetoacetyl-CoA and acetyl-CoA to HMG-CoA will have a positive effect on both IDP production and maximum levels of HMG-CoA. Thus, over-expressing this enzyme by itself will not have significant effects. However, we additionally note that over-expressing *E*_4_, the enzyme that catalyses the reaction from HMG-CoA to mevalonate, will decrease the levels of HMG-CoA without having a significant effect on IDP production. Therefore, over-expressing both *E*_3_ and *E*_4_ should have a much larger effect on IDP production than just over-expressing *E*_3_. The importance of the reaction from HMG-CoA to mevalonate has been previously noted by experiments (Pitera et al., 2007). We also note that increasing *E*_1_ has a positive effect on both IDP production for continuously replenished pyruvate, and on the maximum levels of HMG-CoA. Thus, as for *E*_3_, this effect can be amplified by also over-expressing *E*_4_ at the same time. We illustrate these results in Figs. 6 and 7.

Our results also suggest that the reactions between acetyl-CoA and acetoacetyl-CoA can have a significant effect on both IDP production and maximum levels of HMG-CoA. However, this effect cannot be achieved by over- or under-expressing *E*_2_, the enzyme that catalyses this reaction, as the enzyme catalyses the reaction in both directions. We see that this positive effect can be attained by using an enzyme that strongly favours the forward reaction. That is, an enzyme with a large ratio of $k_{2}K_{-2}^{M}/k_{-2}K_{2}^{M}$. The final reaction that plays an important role at leading order is the reaction from acetyl-CoA to a sink, which has a negative leading-order effect on both IDP production and the maximum levels of HMG-CoA. Although we consider the dimensionless sink coefficient to be of *O*(1) in our main analysis, we also show in Appendix A that a large sink coefficient does not significantly affect the system behaviour. However, we note that the importance of acetyl-CoA to other metabolic pathways represented by this sink, such as the citric acid cycle, means that there are likely to be negative effects to the cell if this reaction is significantly altered. Our results suggest that the reactions and enzymes we have mentioned are the only significant reactions at leading order, and we predict that the over-expression of other enzymes in the pathway will not have a significant effect on either IDP production or on the maximum levels of HMG-CoA.

In the case where pyruvate was never replenished, we found that the depletion of pyruvate occurred slowly enough that, for intermediate time, our system reproduced the steady-state behaviour we would eventually expect from the continuously replenished pyruvate case. However, whilst the system was initially robust to this slow depletion, we additionally calculated the point at which the low levels of pyruvate affected the entire system, then calculated the dynamics of this depletion. This required the use of the method of matched asymptotic expansions with logarithmic matching terms involving the small variable to track metabolite concentrations up to the first correction order over two timescales, thus allowing us to obtain the leading-order depletion at the third and final timescale.

In our experiments, we were able to introduce a pathway from pyruvate to mevalonate into the bacterium *C. necator*. This was achieved by transforming *C. necator* with a plasmid harbouring the *E. faecalis mvaE* and *mvaS* genes under the control of a promoter induced by the presence of *L*-arabinose (P_BAD_). The proteins MvaE and MvaS, respectively encoded by these two genes, are responsible for the conversion of acetyl-CoA to mevalonate. A protein expression analysis showed that production of MvaE increased as the levels of *L*-arabinose present in the cultivation medium were increased. On the other hand, expression of MvaS appeared to be independent of *L*-arabinose concentration. In any case, the effect of increasing MvaE expression was to produce more mevalonate, an experimental result that agreed with the theoretical predictions from our model. We note that our model could be verified further by directly measuring the levels of HMG-CoA. However, as HMG-CoA is not secreted by the cell and is likely to be unstable, direct measurement is significantly more technical; as carried out in Pitera et al. (2007), the experiments would have to be repeated while rapidly quenching the cellular metabolism, before examining the endo-metabolites using mass spectrometry.

In the derivation of this model, we chose initial conditions that modelled the instantaneous addition of pyruvate to a well-mixed solution of enzymes. These conditions were chosen for mathematical convenience and are likely to differ significantly from the conditions within a cell producing isopentenyl-diphosphate. However, as the systems we have considered both tend to stable steady states, this difference is unlikely to be a practical issue for the long time results, although it would affect the initial transients.

Finally, we note that this work highlights how a simple mathematical model can be used to predict the biologically relevant behaviour of a system. Moreover, this work shows how asymptotic analysis can play an important role in reducing the computational complexity of a derived system and be used to overcome uncertainty issues with parameter values. We hope that the predictions from our model can help to build a more efficient path to biological discovery.

## Figures and Tables

**Fig. B.1:**
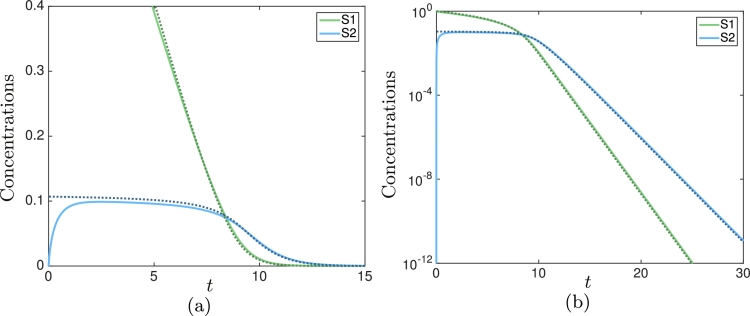
The solutions to the full and reduced systems for the concentrations of *S*_1_ and *S*_2_ in the no replenishment of pyruvate case. We show (a) A linear plot (b) A log-lin plot. The solid light lines denote the numerical solutions to the full problem, and the dashed darker lines denote the solutions to the reduced problem given in (B.8). We see good agreement between the numerical and asymptotic solutions apart from at early time, as we expect.

## Appendix A Large $\bar{A}$ limit

There is another distinguished limit in the system (3) when $\bar{A}=O\left(1/ɛ\right)$. We briefly summarise the system behaviour in this limit for t=O(1). The leading-order system (5) is mainly unchanged, apart from the first equation for ${\bar{S}}_{2},$ which becomes

(A.1) $$\begin{array}{c} \frac{ɛ{\bar{K}}_{1}^{i}S_{1}}{{ɛ}^{1/2}{\bar{K}}_{1}^{i}\left(S_{1}+{\bar{K}}_{1}^{M}\right)+S_{1}S_{2}}=ɛ\bar{A}S_{2}, \end{array}$$

where we now have $S_{2}=O\left({ɛ}^{1/2}\right),$ providing an asymptotic balance between the two terms. Rearranging (A.1) for *S*_2_ in terms of *S*_1_, we obtain

(A.2) $$\begin{array}{c} S_{2}=\frac{{ɛ}^{1/2}}{2}\left[{\left\{{\left(\frac{{\bar{K}}_{1}^{i}\left(S_{1}+{\bar{K}}_{1}^{M}\right)}{S_{1}}\right)}^{2}+\frac{4{\bar{K}}_{1}^{i}}{ɛ\bar{A}}\right\}}^{1/2}-\frac{{\bar{K}}_{1}^{i}\left(S_{1}+{\bar{K}}_{1}^{M}\right)}{S_{1}}\right]. \end{array}$$

For the continuous-replenishment case we have *S*_1_(*t*) ≡ 1, and for the no-replenishment case the governing equation for *S*_1_ is given by (10). Finally, focusing on the continuous-replenishment case, we note that we can re-write (7), the long-time production of IDP, as

(A.3) $$\begin{array}{c} \bar{P}∼\left(\frac{v_{2}v_{3}}{v_{-2}}t\right)\lim_{t\to \infty }S_{2}\left(t\right)\;\text{as}\;t\to \infty . \end{array}$$

Thus, from (A.2) we see that the long-time production of IDP gains some further dependence on the kinetic properties of *E*_1_ in the limit of large $\bar{A},$ but retains the same qualitative behaviour.

## Appendix B Dynamics of pyruvate depletion

In this appendix, we further investigate the dynamics of pyruvate depletion. We only investigate ${\bar{S}}_{1}$ and ${\bar{S}}_{2}$ here, as the leading-order versions of the remaining metabolites can be obtained by suitably rescaling the solutions (6 b–g) for the timescales we present in this appendix.

To obtain the leading-order solutions during the important depletion timescale, we require the long-time behaviour of the *O*(ϵ^1/2^) correction to ${\bar{S}}_{2}$ at t=O(1). To this end, we may use the leading-order solution (6) to write the asymptotic expansion

(B.1) $$\begin{array}{c} {\bar{S}}_{2}∼{\left(\frac{{\bar{K}}_{1}^{i}\left(1-e^{-2\alpha t}\right)}{\alpha }\right)}^{1/2}+{ɛ}^{1/2}{\bar{K}}_{1}^{i}s_{2}\left(t\right)\;\text{as}\;ɛ\to 0, \end{array}$$

where $s_{2}\left(t\right)=O\left(1\right)$ is the first correction term for ${\bar{S}}_{2}$. We combine (4a,b), (11), and (B.1) to obtain

(B.2a) $$\begin{array}{cc} \frac{1}{\alpha }\frac{ds_{2}}{dt}+\left(\frac{2-e^{-2\alpha t}}{1-e^{-2\alpha t}}\right)s_{2} & =\frac{2\beta }{1-e^{-2\alpha t}}+2\gamma \left(1-e^{-2\alpha t}\right), \end{array}$$

where

(B.2b) $$\begin{array}{cc} \beta & =-\frac{{\bar{K}}_{1}^{M}+1}{2}, \end{array}$$

(B.2c) $$\begin{array}{cc} \gamma & =\frac{v_{2}v_{3}}{2\alpha^{2}v_{-2}}\left(\frac{1}{{\bar{K}}_{2}^{M}}-\frac{v_{2}}{{\bar{k}}_{-2}}+\frac{v_{2}v_{3}}{v_{-2}{\bar{k}}_{3}}\right). \end{array}$$

Solving (B.2a) in the long-time limit, we deduce that

(B.3) $$\begin{array}{c} s_{2}∼\beta +\gamma +\text{exponentially}\;\text{small}\;\text{terms}\;\text{as}\;t\to \infty , \end{array}$$

and thus we are able to determine the long-time behaviour

(B.4) $$\begin{array}{c} {\bar{S}}_{2}∼{\left(\frac{{\bar{K}}_{1}^{i}}{\alpha }\right)}^{1/2}+{ɛ}^{1/2}{\bar{K}}_{1}^{i}\left(\beta +\gamma \right)\;\text{as}\;t\to \infty . \end{array}$$

The *O*(1) depletion of pyruvate occurs over the timescale $t=O({ɛ}^{-1/2}),$ and we investigate this using the scaling $t=T/{ɛ}^{1/2},$ where T=O(1). Writing ${\bar{S}}_{1}$ and ${\bar{S}}_{2}$ as the asymptotic expansions

(B.5) $$\begin{array}{c} {\bar{S}}_{1}∼{\bar{S}}_{1}^{\left(0\right)}+{ɛ}^{1/2}{\bar{S}}_{1}^{\left(1\right)},\;{\bar{S}}_{2}∼{\bar{S}}_{2}^{\left(0\right)}+{ɛ}^{1/2}{\bar{S}}_{2}^{\left(1\right)}\;\text{as}\;ɛ\to 0, \end{array}$$

the appropriate governing equations, obtained from (10) and (4a,b), are

(B.6a) $$\begin{array}{cc} \frac{d{\bar{S}}_{1}^{\left(0\right)}}{dT} & =-\frac{{\bar{K}}_{1}^{i}}{{\bar{S}}_{2}^{\left(0\right)}}, \end{array}$$

(B.6b) $$\begin{array}{cc} \frac{d{\bar{S}}_{1}^{\left(1\right)}}{dT} & =\frac{{\bar{K}}_{1}^{i}}{{\left({\bar{S}}_{2}^{\left(0\right)}\right)}^{2}}\left({\bar{S}}_{2}^{\left(1\right)}+{\bar{K}}_{1}^{i}\left(1+\frac{{\bar{K}}_{1}^{M}}{{\bar{S}}_{1}^{\left(0\right)}}\right)\right), \end{array}$$

(B.6c) $$\begin{array}{cc} 0 & =\frac{{\bar{K}}_{1}^{i}}{{\bar{S}}_{2}^{\left(0\right)}}-\alpha {\bar{S}}_{2}^{\left(0\right)}, \end{array}$$

(B.6d) $$\begin{array}{cc} \frac{d{\bar{S}}_{2}^{\left(0\right)}}{dT} & =-2\alpha \left({\bar{S}}_{2}^{\left(1\right)}+{\bar{K}}_{1}^{i}\left(\frac{1}{2}\left(1+\frac{{\bar{K}}_{1}^{M}}{{\bar{S}}_{1}^{\left(0\right)}}\right)-\gamma \right)\right), \end{array}$$

with matching conditions, valid when $T\to 0^{+},$

(B.6e) $$\begin{array}{c} {\bar{S}}_{1}^{\left(0\right)}∼1-{\left(\alpha {\bar{K}}_{1}^{i}\right)}^{1/2}T,\;{\bar{S}}_{1}^{\left(1\right)}∼-{\left(\frac{{\bar{K}}_{1}^{i}}{\alpha }\right)}^{1/2}\log2,\\ {\bar{S}}_{2}^{\left(0\right)}∼{\left(\frac{{\bar{K}}_{1}^{i}}{\alpha }\right)}^{1/2},\;{\bar{S}}_{2}^{\left(1\right)}∼{\bar{K}}_{1}^{i}\left(\beta +\gamma \right), \end{array}$$

obtained using Van Dyke’s matching rule (van Dyke, 1975) on the t=O(1) results (11) and (B.4).

The system (B.6) is thus solved by

(B.7a) $$\begin{array}{cc} {\bar{S}}_{1}^{\left(0\right)} & =1-{\left(\alpha {\bar{K}}_{1}^{i}\right)}^{1/2}T, \end{array}$$

(B.7b) $$\begin{array}{ccc} {\bar{S}}_{1}^{\left(1\right)} & = & \alpha {\bar{K}}_{1}^{i}\left(\gamma +\frac{1}{2}\right)T-\frac{{\bar{K}}_{1}^{M}{\left(\alpha {\bar{K}}_{1}^{i}\right)}^{1/2}}{2}\log\left(1-{\left(\alpha {\bar{K}}_{1}^{i}\right)}^{1/2}T\right)\\ & & -\;{\left(\frac{{\bar{K}}_{1}^{i}}{\alpha }\right)}^{1/2}\log2, \end{array}$$

(B.7c) $$\begin{array}{cc} {\bar{S}}_{2}^{\left(0\right)} & ={\left(\frac{{\bar{K}}_{1}^{i}}{\alpha }\right)}^{1/2}, \end{array}$$

(B.7d) $$\begin{array}{cc} {\bar{S}}_{2}^{\left(1\right)} & ={\bar{K}}_{1}^{i}\left(\beta +\gamma -\frac{{\left(\alpha {\bar{K}}_{1}^{i}\right)}^{1/2}T}{2\left(1-{\left(\alpha {\bar{K}}_{1}^{i}\right)}^{1/2}T\right)}\right). \end{array}$$

The most significant asymptotic regime of interest for the depletion dynamics occurs when ${\bar{S}}_{1}=O\left({ɛ}^{1/2}\right),$ which results in the depletion of several metabolites in the system. This region occurs when $t=1/{\left(ɛ\alpha {\bar{K}}_{1}^{i}\right)}^{1/2}+\hat{t}/\alpha$ (equivalently, when $T=1/{\left(\alpha {\bar{K}}_{1}^{i}\right)}^{1/2}+{ɛ}^{1/2}\left(\hat{t}/\alpha \right)$), where $\hat{t}=O\left(1\right)$. We additionally scale $\left({\bar{S}}_{1},{\bar{S}}_{2}\right)={\left({\bar{K}}_{1}^{i}/\alpha \right)}^{1/2}\left({ɛ}^{1/2}{\hat{S}}_{1}\left(\hat{t}\right),{\hat{S}}_{2}\left(\hat{t}\right)\right)$. Substituting these scalings into (10) and (4a,b) results in the leading-order long-time equations

(B.8a) $$\begin{array}{cc} \frac{d{\hat{S}}_{1}}{d\hat{t}} & =-\frac{{\hat{S}}_{1}}{\alpha {\bar{K}}_{1}^{M}+{\hat{S}}_{1}{\hat{S}}_{2}}, \end{array}$$

(B.8b) $$\begin{array}{cc} \frac{d{\hat{S}}_{2}}{d\hat{t}} & =\frac{{\hat{S}}_{1}}{\alpha {\bar{K}}_{1}^{M}+{\hat{S}}_{1}{\hat{S}}_{2}}-{\hat{S}}_{2}. \end{array}$$

The leading-order matching conditions as $\hat{t}\to -\infty$ are ${\hat{S}}_{1}∼-\hat{t}$ and ${\hat{S}}_{2}\to 1,$ and the remaining variables in the system are still given by (6 c-h). In practice, we find that this leading-order system is sensitive to the *O*(ϵ^1/2^) matching condition for ${\hat{S}}_{1}$ as $\hat{t}\to -\infty$. We can deduce this directly by determining the behaviour of ${\hat{S}}_{1}$ as $\hat{t}\to -\infty$ from the system (B.8). That is, we deduce that

(B.9a) $$\begin{array}{c} {\hat{S}}_{1}∼-\hat{t}-\left(\alpha {\bar{K}}_{1}^{M}/2\right)\log\left(-\hat{t}\right)+C\;\text{as}\;\hat{t}\to -\infty . \end{array}$$

We may determine *C* by matching the solution to ${\bar{S}}_{1}$ up to *O*(ϵ^1/2^) when $t=O({ɛ}^{-1/2}),$ given in (B.7a,b). From this matching, we deduce that

(B.9b) $$\begin{array}{ccc} C & = & -\;\log2+\frac{1}{2}\left(\frac{v_{2}v_{3}}{\alpha v_{-2}}\left(\frac{1}{{\bar{K}}_{2}^{M}}-\frac{v_{2}}{k_{-2}}+\frac{v_{2}v_{3}}{k_{3}v_{-2}}\right)+\alpha \right)\\ & & -\;\frac{\alpha {\bar{K}}_{1}^{M}}{4}\log\left(\frac{ɛ{\bar{K}}_{1}^{i}}{\alpha }\right)+O\left({ɛ}^{1/2}\right). \end{array}$$

Using (B.9) as the matching condition for ${\hat{S}}_{1},$ we may numerically solve (B.8) to obtain the depletion dynamics for ${\hat{S}}_{1}$ and ${\hat{S}}_{2},$ then use the latter solution in (6 b–g) to deduce the depletion dynamics for the remaining metabolites. We see excellent agreement between these asymptotically determined solutions and the full numerical solutions (Fig. B.1).

As the system (B.8) is governed by two coupled autonomous nonlinear equations, we further note that we are able to reduce these to a single nonlinear ordinary differential equation by dividing one equation by the other and seeking a solution to ${\bar{S}}_{2}\left({\bar{S}}_{1}\right)$. Although this first-order differential equation can be transformed into a linear second-order differential equation with solutions involving modified Bessel functions, reintroducing *t* into this system is nontrivial, and the solutions do not provide any insight into the underlying system behaviour. Therefore, we do not consider this route further.
